# Adverse events associated with long-term ketamine use in pediatric septic shock

**DOI:** 10.1186/cc10157

**Published:** 2011-06-22

**Authors:** CMF Mangia, AFCF Martins, AP Loretti, RM Sousa, MC Andrade

**Affiliations:** 1 Universidade Federal de São Paulo, São Paulo - SP, Brazil

## Objective

Ketamine hydrochloride is a noncompetitive antagonist of the NMDA receptors and produces a dissociative state described as a 'functional and neuro-physiological dissociation between the neocortical and limbic systems' [1,2].

## Methods

We describe long-term use of ketamine in the pediatric intensive care unit (PICU) inducing pyramidal liberation in a septic shock patient.

## Case

A 15-month-old boy with congenital cardiopathy and developmental delay without previous chronic encephalopathy history. He was admitted with septic shock and during the PICU stay received association of multiple analgesic-sedative agents and high doses of ketamine intravenous infusion (Figure 1). The patient presented after 10 days of PICU stay symptoms associated with pyramidal liberation: deep hyperreflexia with sinreflexia, Babinski sign on both sides, opisthotonus, trismus. The clinical signs were not associated with new metabolic or structural intracranial lesion. The patient was discharged from hospital after 36 days receiving pericyazine that was interrupted 1 week after hospital discharge.

## Conclusion

The ketamine side effects after short-term use include [1,2]: hypertension, apnoea, laryngospasm, emergence phenomena, vomiting, nystagmus, ataxia, myoclonus, random limb movements, opisthotonus, transient facial rash or flushing, intracranial hypertension. The long-term-use side effects are unknown. This is the first report of pyramidal liberation-associated intravenous ketamine for a prolonged period.
